# At-Risk Populations for Osteosarcoma: The Syndromes and Beyond

**DOI:** 10.1155/2012/152382

**Published:** 2012-03-12

**Authors:** George T. Calvert, R. Lor Randall, Kevin B. Jones, Lisa Cannon-Albright, Stephen Lessnick, Joshua D. Schiffman

**Affiliations:** ^1^Department of Orthopaedics and Huntsman Cancer Institute, The University of Utah, Salt Lake City, UT 84112, USA; ^2^Sarcoma Services, Center for Children, Huntsman Cancer Institute, The University of Utah, Salt Lake City, UT 84112, USA; ^3^Division of Genetic Epidemiology, Department of Biomedical Informatics, University of Utah School of Medicine, Salt Lake City, Utah 84108, USA; ^4^Division of Pediatric Hematology Oncology, Department of Pediatrics, The University of Utah, Salt Lake City, UT 84112, USA

## Abstract

Osteosarcoma is the most common primary malignancy of bone. Most cases are sporadic without a known genetic or environmental cause. Heritable genetic predisposition syndromes are associated with a small percentage of osteosarcomas. Study of these rare disorders has provided insight into the molecular pathogenesis of osteosarcoma. Screening of at-risk families and surveillance of affected individuals for these syndromes may permit earlier diagnosis and more effective treatment of osteosarcoma in these populations. This paper reviews the genetic and clinical features of the known osteosarcoma predisposition syndromes.

## 1. Introduction

Osteosarcoma is the most common primary malignant bone tumor. It has a bimodal age distribution with the larger peak among adolescents and young adults and a second smaller peak among the elderly [1]. The majority of osteosarcoma cases are sporadic with no identifiable genetic or environmental cause [2]. A small and unknown percentage of osteosarcomas occur in individuals with cancer predisposition syndromes. A review of the Dutch National Pathology Register identified secondary malignancies in 7% of osteosarcoma patients, indicating possible hereditary cancer syndromes in these patients [3]. A nationwide study from Sweden found significant associations between childhood osteosarcoma and several parental cancers including rectal cancer, colon cancer, endocrine cancers, melanoma, and breast cancer [4].

 The known osteosarcoma predisposition syndromes include Li-Fraumeni syndrome (LFS), hereditary retinoblastoma (RB), Rothmund-Thomson syndrome (RTS) type 2, Werner syndrome (WS), Bloom syndrome (BS), RAPADILINO syndrome, and Diamond Blackfan anemia (DBA). The epidemiology, clinical findings, genetics, treatment, and screening recommendations for these syndromes will be reviewed. Additionally, insights into the pathogenesis of osteosarcoma derived from these syndromes will be discussed.

## 2. Li-Fraumeni Syndrome (*TP53*)

### 2.1. Description and Diagnosis

 In 1969, Li and Fraumeni described a clinical phenotype and syndrome associated with soft tissue sarcomas, breast cancer, and other cancers [5]. Germline mutations of the *TP53 *gene were first identified among familial cohorts with LFS in 1990 (Table 1) [6, 7]. *TP53* acts as a cell cycle regulator, and mutations of *TP53* are found in approximately 50% of human cancers [8]. This mutation has subsequently been identified as causative in approximately 70% of individuals with LFS [9, 10]. Another gene, *CHEK2*, was initially believed to be the responsible gene in some of the LFS families who did not have *TP53* mutations. Subsequent research suggests that this association is unlikely [11, 12]. LFS is inherited as an autosomal dominant disease and has been identified among cohorts throughout the world. Diagnosis of LFS is based upon clinical criteria as not all families with a classic presentation carry the *TP53* mutation. In addition to families with “classic LFS,” there are also numerous reported families who have abnormally high rates of cancers and increased rates of *TP53* mutations. These cohorts have been diagnosed with Li-Fraumeni-like (LFL) syndrome. Published criteria for LFS and LFL syndrome are listed in Table 2 [13, 14].

Sarcomas, premenopausal breast cancer, brain tumors, and adrenocortical carcinomas account for the majority of cancers among LFS and LFL patients and are considered the “classic” Li-Fraumeni cancers. However, almost every type of known cancer is overrepresented in this population including lung cancer, leukemia, lymphoma, stomach cancer, colon and rectal cancer, ovarian cancer, and melanoma [15]. Osteosarcoma is the most prevalent bone sarcoma in LFS, but chondrosarcomas have also been reported. In a review of 24 families, 18 of 151 affected individuals developed osteosarcoma. There were three chondrosarcomas in the same study [13]. Ewing sarcoma has not been associated with LFS or any other hereditary cancer syndrome [15]. The presentation of osteosarcoma among LFS patients is similar to sporadic osteosarcoma with respect to location. Age at presentation of LFS-associated osteosarcomas is younger than in the general population [16]. Some authors have suggested that genetic anticipation and progressive shortening of telomere length is at least partially responsible for this finding [17]. Although LFS and germline *TP53* mutations are rare in the population, the *TP53* gene is frequently mutated in tumor samples from a variety of cancers in non-LFS patients including osteosarcoma. Somatic *TP53* mutations are identified in 15–30% of all osteosarcoma specimens with the variation depending on the testing methods and study population used [18, 19].

### 2.2. Treatment and Screening for LFS

 Cancers which occur in individuals with LFS are generally treated with standard therapy for the specific cancer in question. The major exception to this principle is that radiation exposure for LFS patients should be minimized if possible [20]. For example, mastectomy is often recommended for breast cancer in LFS patients in order to avoid the need for adjuvant radiotherapy. A recent report from France reviewed eight LFS patients with breast cancer as their first diagnosis. Three of the eight received conservative breast surgery and radiation instead of mastectomy, and an additional three patients received radiation therapy in addition to mastectomy. All three lumpectomy patients developed recurrent local disease. Additionally, there were two radiation-induced sarcomas among the six patients who received radiation [21]. Radiotherapy does not play as large of a role in the treatment of osteosarcoma. However, chest computed tomography (CT) and bone scans are routinely used for preoperative staging and posttreatment surveillance of osteosarcoma patients. Current surveillance guidelines require as many as 15 posttreatment chest imaging studies (NCCN, Bone Cancer, Version 2, 2011). Use of chest radiographs instead of CT scans decreases radiation exposure in the LFS population. Whole body magnetic resonance imaging (MRI) has been proposed as a screening and surveillance imaging alternative which completely avoids the use of radiation in this population [22]. In fact, a recent study showed that annual whole body MRI combined with blood tests for common LFS cancers can facilitate early detection of cancers and decrease mortality of LFS patients [23]. The National Comprehensive Cancer Network (NCCN) guidelines for cancer screening among LFS patients are listed in Table 3. Other NCCN management recommendations include avoidance of radiation therapy if possible and consideration of prophylactic mastectomies in some high-risk patients.

 Genetic counseling and testing should be offered to individuals at high risk of having germline *TP53* mutations. These include family members of individuals known to have germline *TP53* mutations or who meet classic LFS criteria. Additionally, Chompret et al. developed *TP53* testing criteria [24] to identify mutations among individuals and families not meeting classic LFS criteria (Table 2). These criteria were recently updated to include testing of any individual, regardless of age, with a history of adrenocortical carcinoma or choroid plexus carcinoma [25]. Another recent study further validated the 2009 Chompret criteria and additionally supported the testing of breast cancer patients under the age of 30 years if *BRCA1/BRCA2* mutations are not identified [26]. Some geneticists will also test for *TP53* mutations in osteosarcoma patients presenting before 10 years of age.

## 3. Hereditary Retinoblastoma

### 3.1. Description and Diagnosis

Hereditary retinoblastoma is a rare, autosomal dominant disease which causes malignant eye tumors in children and an increased risk of subsequent malignancies, especially osteosarcomas, later in life. Retinoblastomas, the primary tumors involved in the disease, arise from developing retinal tissue in young children. The retinoblastoma cell of origin has not been fully defined [27]. Although rare, retinoblastoma is the most common intraocular cancer with an incidence of 1 in 16,000 to 1 in 18,000 live births [28]. Retinoblastoma affects all races and geographic regions. Retinoblastoma is one of the most treatable pediatric cancers with 5-year actuarial survival rates in the USA over 95% between 1995 and 2004 [29]. The diagnosis of retinoblastoma is based upon clinical eye exams. Young, at-risk patients may require exam under anesthesia to confirm the diagnosis. Modern treatment for retinoblastoma, even for the heritable form, is often less morbid than in the past as enucleation of the eye often can be avoided with the use of chemotherapy [30].

Heritable retinoblastoma is caused by a mutation of the *RB1* tumor suppressor gene. The study of heritable retinoblastoma has provided fundamental insights into cancer biology. In 1971, Albert Knudson proposed the “two-hit hypothesis” of cancer development based upon statistical analysis of hereditary and spontaneous retinoblastoma cases [31]. Fundamentally, the hypothesis states that cancer results from accumulated mutations, a “first hit” followed by a “second hit.” In retinoblastoma, cancer develops when a second spontaneous *RB1* mutation occurs in addition to the germline mutation. The hypothesis was confirmed in 1986 when *RB1* became the first cancer susceptibility gene identified in humans [32]. Approximately 42% of retinoblastoma cases were heritable in a large cohort from the United Kingdom [33]. The *RB1* gene product functions as a cell cycle regulator. Homozygous deficiency of *RB1 *is lethal [34].

Similar to *TP53*, somatic mutations of *RB1* are frequently found in tumor specimens from sporadic cases of osteosarcoma and many other cancers. Somatic mutations of *RB1* are found in 30–75% of osteosarcoma specimens depending on the testing techniques utilized and the population studied [35]. Nearly all osteosarcomas are felt to have the RB1 pathway disrupted, some tumors accomplishing this with p16/INK4A silencing or CDK4/Cyclin D1 overexpression [36–38]. In addition to osteosarcoma, hereditary retinoblastoma predisposes individuals to soft tissue sarcomas (especially leiomyosarcoma), melanoma, and brain tumors. There is also a small increase in overall carcinoma rates (especially lung, breast, and bladder) compared to background rates of the general population [39–41]. We continue to learn about more cancers in patients with inherited retinoblastoma as this population now ages.

Osteosarcoma is the most common secondary malignancy in patients with hereditary retinoblastoma. Determining the epidemiology and natural history of osteosarcoma in patients with retinoblastoma was initially complicated by the widespread use of radiation therapy for primary retinoblastoma treatment. A 1997 study of 1,604 retinoblastoma patients diagnosed between 1914 and 1984 revealed a 400-fold increased risk of osteosarcoma among hereditary retinoblastoma patients [40]. Much of this increased risk resulted from the combination of genetic susceptibility and the use of radiation therapy. The same authors noted that both bone and soft tissue sarcoma rates were increased by radiation exposure in a dose-dependent fashion. Moreover, 69% of the osteosarcomas in the study occurred in the head. Fletcher et al. reported a British hereditary retinoblastoma cohort of patient born between 1873 and 1950 when external beam radiation was infrequently used for retinoblastoma [42]. Only 8 of 144 (5.5%) patients developed sarcomas of any type, while the overall incidence of secondary malignancy remained high. Another study from the United Kingdom identified a 200-fold increased rate of osteosarcoma among 809 heritable retinoblastoma patients diagnosed between 1951 and 2004 [43]. The same study also concluded that the osteosarcomas generally occurred in adolescents and young adults, similar to sporadic cases. Radiation use, radiation dosage, and osteosarcoma site were not reported in this study. However, it is important to remember that even patients with hereditary retinoblastoma who did not receive radiation exposure are still at increased risk for the development of osteosarcoma due to their underlying RB mutation.

### 3.2. Treatment and Screening

 Treatment of primary disease among hereditary retinoblastoma patients has evolved over the past several decades. External beam radiation is used less frequently and in lower doses. Enucleation rates are lower and vision is preserved more frequently than in the past [44]. Retinoblastoma patients with osteosarcoma are treated with standard chemotherapy protocols. One 2003 study from Spain suggested that osteosarcoma patients with loss of heterozygosity of *RB1 *had worse prognosis [45]. This has not been confirmed among other cohorts. Similar to LFS patients, ionizing radiation exposure of retinoblastoma patients should be limited if possible due to the risk of radiation-induced sarcomas. Unlike LFS, there are no established surveillance guidelines for secondary malignancies among retinoblastoma survivors [46]. Patient and family education should emphasize avoidance of environmental carcinogens such UV radiation, ionizing radiation, and tobacco use. Heightened concern for lingering musculoskeletal symptoms or unexplained masses is prudent due to the increased incidence of sarcomas. Increased breast health vigilance (self-exam, clinical exam, and mammography) has also been advocated by some authors due the limited but observed increase in breast cancer among retinoblastoma patients [47, 48].

 Genetic testing for *RB1* mutations is routinely offered to affected individuals and their families. Testing is valuable for distinguishing sporadic from familial cases. The American Society of Clinical Oncology recommends that genetic testing should be part of the standard treatment for individuals and first-degree relatives with retinoblastoma cancer predisposition disorder [49]. Genetic testing is strongly recommended because intensive surveillance of newborns with the mutation can lead to early detection and more effective, vision sparing treatment. Overall survival is increased and enucleation rates are decreased with early detection [50]. Intensive screening requires eye examinations every 3 to 4 weeks until age three. This regimen often requires repeated examinations under anesthesia. This regimen can be avoided in unaffected family members identified with the use of genetic testing. The possibility of somatic and germline mosaicism must also be considered when recommending genetic testing and interpreting its results. A small percentage (approximately 5%) of unilateral retinoblastoma patients with no family history of the disease develop contralateral retinoblastomas despite having negative blood leukocyte tests for *RB1* mutations [51]. Some individuals with the mutation will have a less than a fifty percent chance of transmitting the disease due to germline mosaicism. The added complexity of mosaicism emphasizes the importance of referral to genetic counseling in addition to testing.

## 4. *RecQ* Disorders Associated with Osteosarcoma Predisposition

### 4.1. Introduction

 DNA helicases are proteins which aid in the unwinding of double-stranded DNA during replication and repair. There are several different helicases, and the function of many is not yet fully elucidated. Four very rare syndromes associated with osteosarcoma predisposition are caused by mutations of the *RecQ* family of helicase genes. There are five known members of the *RecQ* family in humans, three of which have been associated with syndromes [52]. Unlike LFS and retinoblastoma, the helicase syndromes are autosomal recessive and have distinct physical findings.  Table 4 lists the most common phenotypic findings of the *RecQ* osteosarcoma predisposition syndromes. Of the four *RecQ* syndromes, Rothmund-Thomson Syndrome is most strongly associated with osteosarcoma predisposition. Overlapping findings of the four syndromes include chromosomal instability, growth retardation, dermatological changes, and cancer predisposition. In contrast to *TP53* and *RB *mutations, the *RecQ* mutations have not been commonly found in somatic tissue from sporadic cancer cases [52, 53]. Silencing of *RecQ* expression through methylation has been proposed as a mechanism by which these genes may be involved in sporadic cancer without detectable mutations [53].

### 4.2. Rothmund-Thomson Syndrome (*RecQL4*)

 Rothmund first described a syndrome of poikiloderma, growth retardation, and bilateral cataracts among an inbred family in Bavaria in 1868. Thomson, in 1923, reported a group of patients with poikiloderma, growth retardation, and skeletal abnormalities but without cataracts. In 1957, Taylor identified the two cohorts as the same syndrome [54]. Patients with Rothmund-Thomson Syndrome (RTS) usually present with erythema, swelling, and blistering of the face and extremities between the age of 3 and 6 months. Mutations of *RecQ4* (also known as *RecQL4)* were identified as the cause of some cases of RTS in 1999 [55]. Subsequent research has identified two types of RTS. Type 1 RTS patients lack *RecQ4* mutations, develop cataracts, and are not predisposed to osteosarcomas. The gene responsible for Type 1 RTS is unknown at this time. Type 2 RTS patients have *RecQ4* mutations, a greater number and severity of skeletal abnormalities, and a predisposition to osteosarcomas [56]. *RecQ4* is unique among the *RecQ *family in that it lacks detectable DNA helicase activity and possesses single-stranded DNA annealing activity [57]. Researchers hypothesize that* RecQ4* functions in DNA repair.

 A 2008 review of the world literature identified 61 cases of cancer among all reported RTS patients, of which 38 (62%) were osteosarcomas. Three cases were multicentric (metachronous) osteosarcoma, and 12 cases developed before age 10, with the overall average age of presentation at 14 years [58]. Another cohort of 41 RTS patients contained a 31% incidence of osteosarcoma with a mean age of 11.5 years and 4 multicentric cases [54]. The same authors recommended obtaining baseline long bone radiographs by age five in RTS patients. They recommended this imaging in order to identify subtle skeletal dysplasia findings as well as to provide a baseline comparison if the patients develop musculoskeletal symptoms concerning for osteosarcoma later in life. A 2003 study of 33 RTS patients found an association between gene truncation (as opposed to nonsense or missense) mutations and osteosarcoma development [59]. The authors proposed that this genotype/phenotype correlation may be useful in identifying RTS patients at increased risk of developing osteosarcoma.

### 4.3. RAPADILINO Syndrome (*RecQ4*)

 A second syndrome associated with osteosarcoma and caused by *RecQ4* mutations has only recently been described. The name is an acronym of the major clinical findings (RA: RAdial aplasia or hypoplasia, PA: PAtellae aplasia or hypoplasia and cleft or high arched PAlate, DI: DIarrhea and DIslocated joints, LI: LIttle size and LImb malformations, NO: long, slender NOse and NOrmal intelligence). Notably, the hallmark poikiloderma of RTS is absent among RAPADILINO patients. A 1989 report from Finland first identified the syndrome in 5 patients [60]. The disease is extremely rare and predominantly found in Finland although cases in other parts of the world have been reported. In 2003, *RecQ4* mutations were identified as the cause [61]. Osteosarcoma was not initially thought to be associated with the condition; however, a 2009 study found that 2 of 15 (13.3%) known cases in Finland developed osteosarcoma [62]. Based upon these data, RAPADILINO should be included in the list of osteosarcoma predisposition syndromes. Baller-Gerold syndrome, the third condition associated with mutation of *RecQ4*, is characterized by radial aplasia and craniosynostosis. It has not been associated with osteosarcoma [62].

### 4.4. Werner Syndrome (*WRN*)

 Werner syndrome (WS), also known as adult progeria, is a rare autosomal recessive condition characterized by premature aging, short stature, bilateral cataracts, and distinctive skin changes [63]. The condition is overrepresented in Japan, presumably due to a founder effect similar to RAPADILINO syndrome. Diagnosis is often not made until the fourth decade of life [64]. WS is caused by mutations of the *WRN *gene which was first cloned in 1996 [65]. The *WRN* gene product is the only human *RecQ* family member with 3′ to 5′ exonuclease activity. The relevance of this finding to the unique progeria phenotype present in WS has not been determined.

 In a 1996 review of the world literature, osteosarcomas, soft tissue sarcomas, myeloid disorders, benign meningiomas, thyroid cancers, and melanomas were overrepresented in WS. Thirteen of 186 (7%) cancers among WS patients were osteosarcomas [66]. A subsequent study of Japanese WS patients found that the presentation of osteosarcoma in their cohort was atypical. The WS patients developed osteosarcomas at an older age, ranging from 35 to 57. Seven of 10 cases presented in the foot and ankle while one other case presented in the patella (rather than in the long bones typical for osteosarcoma). Half of the osteosarcoma patients also developed additional cancers including three soft tissue sarcomas and three thyroid cancers [67]. Osteosarcomas are treated with standard therapies in WS, and there is no consensus about avoidance of radiation. Because WS is often diagnosed later in life, development of late-onset osteosarcoma may be a presenting complaint of the syndrome.

### 4.5. Bloom Syndrome (*BLM*)

 Bloom syndrome (BS) was first described in 1954. It is characterized by short stature, sun-sensitive rash, and sparseness of subcutaneous fat throughout infancy and early childhood. Mutations of the *BLM* gene were identified as the cause in 1995 [68]. The same study identified *BLM* as a *RecQ* homologue, the first homologue to be identified in humans despite being rarer than RTS and WRN. BS primarily affects Ashkenazi Jews due to a founder effect, but cases have been identified in other ethnic groups, as well [69]. In addition to cancer predisposition, feeding difficulties during infancy and susceptibility to ear and respiratory infections are common clinical problems.

 A review of the first 100 cancers among patients followed in Bloom registry was published in 1997. BS differed from the other *RecQ* syndromes in that the majority (95%) of the cancers were common carcinomas, leukemias, and lymphomas found in the general population [70]. However, the BS registry also contained two osteosarcomas, two Wilm's tumors, and one medulloblastoma. Although the incidence of osteosarcoma in BS is not as high as the previously described syndromes, it far exceeds the expected rate in the general population. Genetic testing for *BLM* mutations is clinically available. The heterozygosity rate among Ashkenazi Jews in New York City is approximately 1%, and testing for BLM mutations is therefore encouraged in this population [71]. Osteosarcomas among BS patients are treated with standard chemotherapy regimens. Screening includes routine physical examinations and earlier initiation of colon cancer screening protocols.

### 4.6. Diamond Blackfan Anemia (*S19* and Other Ribosomal Proteins)

 Diamond Blackfan Anemia (DBA) was first described in 1938 and is characterized by severe pure red blood cell aplasia diagnosed at less than one year of age, frequent congenital abnormalities, and a predisposition to cancer. The condition is clinically and genetically heterogeneous. The North American registry had 420 patients as of 2006, and the estimated incidence of DBA is between 1 in 100,000 and 1 in 200,000 live births [72]. Although rare, DBA is more common than the previously described *RecQ* syndromes. The male-to-female ratio is equivalent, and DBA rates are similar in different countries [73]. Most patients are diagnosed early in infancy, but there are cases of adult diagnosis due to incomplete penetrance of the condition [73]. Congenital anomalies are found in 35–47% of DBA patients, and commonly include craniofacial (50% of all defects), thumb, heart, and renal abnormalities [73]. Treatment of the anemia may include corticosteroids, transfusions, and bone marrow transplantation. Approximately 50% of DBA cases have a known genetic mutation [74]. Ribosomal protein *S19* was the first causative gene to be cloned in 1999 [75]. Subsequently, mutations of eight more ribosomal protein genes were identified as causes of DBA.

 Of the approximately 700 DBA cases reported in the literature, 29 developed cancer [73]. Hematologic malignancies were most common, but six cases of osteosarcoma have been reported, more than would be expected in the general population [76]. Standard treatment protocols for osteosarcoma are recommended in DBA; however, all three cases of osteosarcoma reported in the North American DBA registry had difficulty completing chemotherapy due to cytopenia presumably related to their DBA. The limited number of patients with both conditions precludes analysis of whether having underlying DBA affects survival. There are no formal osteosarcoma surveillance protocols currently recommended for DBA other than close vigilance for bone pain and skeletal masses.

## 5. Summary and Future Direction

Osteosarcoma may be the presenting diagnosis in several of the hereditary syndromes described above including LFS, RTS, WS, and BS. Clinicians who treat osteosarcoma should always consider the possibility of an associated predisposition syndrome, especially in cases of osteosarcoma with an unusual presentation or in patients with a family history of cancer or congenital malformations. A 2005 study from the Netherlands identified definite or suspected malformation syndromes in 7.2% of an unselected cohort of 1,073 pediatric cancer patients [77]. Most notably, 20 of the 42 definite syndrome diagnoses were not identified prior to evaluation for the study. The authors concluded that all children with malignancy may benefit from examination by a clinical geneticist or pediatrician skilled in clinical morphology. Early identification of patients with predisposition syndromes permits treatment of associated pathologies, better cancer surveillance, and genetic testing of at-risk family members. A recent prospective study of 33 patients with germline *TP53* mutations compared intensive and standard surveillance for associated cancers [78]. Intensive surveillance included annual whole body MRI, annual brain MRI, and blood tests every four months. Solid tumors among the intensive surveillance group were identified at an early stage and were surgically resectable. The intensive surveillance group had statistically significant lower mortality at average 2 year followup.

The syndromes reviewed in this paper are rare and often difficult to diagnose unless the clinician has a high index of suspicion. Once identified, patients with these hereditary conditions require frequent and intense medical followup accompanied by referral to a cancer genetics clinic. Progress in the treatment of these conditions has been facilitated by timely and accurate reporting of cases in the literature. National and international registries exist for most of these syndromes to facilitate their study and treatment, and clinicians should enroll their patients in these registries. Patients with osteosarcoma predisposition syndromes are best served by treatment at centers familiar with these rare syndromes. Multidisciplinary care of these patients and their families requires sophisticated genetic testing and counseling in addition to treatment of the disease. Although rare, the study of these hereditary syndromes has provided fundamental insights into cancer biology in general and the pathogenesis of osteosarcoma in particular.

## Figures and Tables

**Table 1:** Osteosarcoma predisposition syndromes and their associated genes.

Syndrome	Inheritance	Genes	Gene product	Chromosome
Li-Fraumeni	AD	*TP53*	Tumor suppressor	17p13.1
Retinoblastoma	AD	*RB1*	Tumor suppressor	13q14.2
Rothmund Thomson II	AR	*REQ4*	DNA helicase	8q24.3
RAPADILINO	AR	*REQ4*	DNA helicase	8q24.3
Werner	AR	*WRN*	DNA helicase	8p12
Bloom	AR	*BLM*	DNA helicase	15q26.1
Diamond Blackfan	AD	*RPS19, RPL5, RPL11, RPL35A, * *RPS24, RPS17, RPS7, RPS10, RPS26*	Ribosomal Protein	Multiple

**Table 2:** Li-Fraumeni diagnosis and screening criteria.

*Classic Li-Fraumeni Criteria*
Proband diagnosed with a sarcoma before age 45
First-degree relative with any cancer before age 45
First- or second-degree relative with any cancer before age 45 or a sarcoma at any age

*Li-Fraumeni-like Criteria*
Proband with any childhood cancer, sarcoma, brain tumor, or adrenocortical carcinoma before age 45
First- or second degree relative with sarcoma, brain tumor, adrenocortical carcinoma, or leukemia at any age
First- or second-degree relative with any cancer before age 60

*2009 Chompret Criteria for TP53 testing*
(I) Proband with tumor belonging to LFS tumor spectrum (e.g., soft tissue sarcoma, osteosarcoma, brain tumor, premenopausal breast cancer, adrenocortical carcinoma, leukemia, and lung bronchoalveolar cancer) before age 46 years and at least one first- or second-degree relative with LFS tumor (except breast cancer if proband has breast cancer) before age 56 years or with multiple tumors; or
(II) Proband with multiple tumors (except multiple breast tumors), two of which belong to LFS tumor spectrum and first of which occurred before age 46 years; or
(III) Patient with adrenocortical carcinoma or choroid plexus tumor, irrespective of family history

**Table 3:** NCCN Li-Fraumeni screening guidelines.

Breast self-exam training and education starting at age 18 years
Clinical breast exam, every 6–12 months, starting at age 20–25 years or 5–10 years before the earliest known breast cancer in the family (whichever comes first).
Annual mammogram or breast MRI starting at age 20–25 years or individualized based upon the earliest age of onset in the family.
Annual comprehensive physical exam; include careful skin and neurologic examinations
Consider colonoscopy every 2–5 years starting no later than age 25 years.
Targeted surveillance based on individual family histories
Discuss options to participate in novel screening approaches using technologies such as PET, abdominal ultrasound, and brain MRI within clinical trials when possible.
Recommend genetic counseling and consideration of genetic testing for at-risk relatives.

**Table 4:** Physical findings of* RecQ* syndromes.

Syndrome	Cutaneous	Craniofacial	Musculoskeletal	Solid organ	Other
RTS II	Poikiloderma Sparse Hair Dystrophic Nails	Frontal bossing Saddle nose	Short Stature Radial Defects Hypoplastic Patellae	Esophageal or Pyloric atresia Annular pancreas	Myelodysplasi Cataracts
RAPADILINO	No Poikiloderma	Slender Nose Cleft Palate	Short Stature Radial Defects Hypoplastic Patellae Joint Dislocations	Diarrhea	Normal intelligence
Werner	Tight, atrophic skin Premature Graying	Bird-like Face	Short Stature Flat Feet Osteoporosis	Hypogonadism	Cataracts Diabetes Mellitus Atherosclerosis
Bloom	Sun Sensitive Rash Telangiectasias	Beaked Nose Narrow Face	Short Stature	Lung disease	High Pitch Voice Mental retardation

## References

[1] Mirabello L, Troisi RJ, Savage SA (2009). International osteosarcoma incidence patterns in children and adolescents, middle ages and elderly persons. *International Journal of Cancer*.

[2] Savage SA, Mirabello L (2011). Using epidemiology and genomics to understand osteosarcoma etiology. *Sarcoma*.

[3] Hauben EI, Arends J, Vandenbroucke JP, van Asperen CJ, Van Marck E, Hogendoorn PCW (2003). Multiple primary malignancies in osteosarcoma patients. Incidence and predictive value of osteosarcoma subtype for cancer syndromes related with osteosarcoma. *European Journal of Human Genetics*.

[4] Ji J, Hemminki K (2006). Familial risk for histology-specific bone cancers: an updated study in Sweden. *European Journal of Cancer*.

[5] Li FP, Fraumeni JF (1969). Soft-tissue sarcomas, breast cancer, and other neoplasms. A familial syndrome?. *Annals of Internal Medicine*.

[6] Srivastava S, Zou Z, Pirollo K, Blattner W, Chang EH (1990). Germ-line transmission of a mutated p53 gene in a cancer-prone family with Li-Fraumeni syndrome. *Nature*.

[7] Malkin D, Li FP, Strong LC (1990). Germ line p53 mutations in a familial syndrome of breast cancer, sarcomas, and other neoplasms. *Science*.

[8] Royds JA, Iacopetta B (2006). p53 and disease: when the guardian angel fails. *Cell Death and Differentiation*.

[9] Birch JM, Hartley AL, Tricker KJ (1994). Prevalence and diversity of constitutional mutations in the p53 gene among 21 Li-Fraumeni families. *Cancer Research*.

[10] Garber JE, Goldstein AM, Kantor AF, Dreyfus MG, Fraumeni JF, Li FP (1991). Follow-up study of twenty-four families with Li-Fraumeni syndrome. *Cancer Research*.

[11] Evans DG, Birch JM, Narod SA (2008). Is CHEK2 a cause of the Li-Fraumeni syndrome?. *Journal of Medical Genetics*.

[12] Ruijs MWG, Broeks A, Menko FH (2009). The contribution of CHEK2 to the TP53-negative Li-Fraumeni phenotype. *Hereditary Cancer in Clinical Practice*.

[13] Li FP, Fraumeni JF, Mulvihill JJ (1988). A cancer family syndrome in twenty-four kindreds. *Cancer Research*.

[14] Birch JM, Hartley AL, Tricker KJ (1994). Prevalence and diversity of constitutional mutations in the p53 gene among 21 Li-Fraumeni families. *Cancer Research*.

[15] Olivier M, Goldgar DE, Sodha N (2003). Li-fraumeni and related syndromes: correlation between tumor type, family structure, and TP53 genotype. *Cancer Research*.

[16] Ognjanovic S, Oliver M, Bergemann TL, Hainaut P Sarcomas in TP53 germline mutation carriers: a review of the IARC TP53 database.

[17] Tabori U, Malkin D (2008). Risk stratification in cancer predisposition syndromes: lessons learned from novel molecular developments in Li-Fraumeni syndrome. *Cancer Research*.

[18] Choong PFM, Broadhead ML, Clark JCM, Myers DE, Dass CR (2011). The molecular pathogenesis of osteosarcoma: a review. *Sarcoma*.

[19] Wang LL (2005). Biology of osteogenic sarcoma. *Cancer Journal*.

[20] Evans DGR, Birch JM, Ramsden RT, Sharif S, Baser ME (2006). Malignant transformation and new primary tumours after therapeutic radiation for benign disease: substantial risks in certain tumour prone syndromes. *Journal of Medical Genetics*.

[21] Heymann S, Delaloge S, Rahal A (2010). Radio-induced malignancies after breast cancer postoperative radiotherapy in patients with Li-Fraumeni syndrome. *Radiation Oncology*.

[22] Monsalve J, Kapur J, Malkin D, Babyn PS (2011). Imaging of cancer predisposition syndromes in children. *Radiographics*.

[23] Villani A, Tabori U, Schiffman J (2011). Biochemical and imaging surveillance in germline TP53 mutation carriers with Li-Fraumeni syndrome: a prospective observational study. *The Lancet Oncology*.

[24] Chompret A, Abel A, Stoppa-Lyonnet D (2001). Sensitivity and predictive value of criteria for p53 germline mutation screening. *Journal of Medical Genetics*.

[25] Tinat J, Bougeard G, Baert-Desurmont S (2009). 2009 version of the Chompret criteria for Li Fraumeni syndrome. *Journal of Clinical Oncology*.

[26] Weitzel JN, Gonzalez KD, Noltner KA (2009). Beyond li fraumeni syndrome: clinical characteristics of families with p53 germline mutations. *Journal of Clinical Oncology*.

[27] Dyer MA, Bremner R (2005). The search for the retinoblastoma cell of origin. *Nature Reviews Cancer*.

[28] Kivelä T (2009). The epidemiological challenge of the most frequent eye cancer: retinoblastoma, an issue of birth and death. *British Journal of Ophthalmology*.

[29] Broaddus E, Topham A, Singh AD (2009). Survival with retinoblastoma in the USA: 1975–2004. *British Journal of Ophthalmology*.

[30] Shields CL, Shields JA (2010). Retinoblastoma management: advances in enucleation, intravenous chemoreduction, and intra-arterial chemotherapy. *Current Opinion in Ophthalmology*.

[31] Knudson AG (1971). Mutation and cancer: statistical study of retinoblastoma. *Proceedings of the National Academy of Sciences of the United States of America*.

[32] Friend SH, Bernards R, Rogelj S (1986). A human DNA segment with properties of the gene that predisposes to retinoblastoma and osteosarcoma. *Nature*.

[33] MacCarthy A, Bayne AM, Draper GJ (2009). Non-ocular tumours following retinoblastoma in Great Britain 1951 to 2004. *British Journal of Ophthalmology*.

[34] Jones KB (2011). Osteosarcomagenesis: modeling cancer initiation in the mouse. *Sarcoma*.

[35] Ottaviani G, Jaffe N (2009). The etiology of osteosarcoma. *Cancer Treatment and Research*.

[36] Nielsen GP, Burns KL, Rosenberg AE, Louis DN (1998). CDKN2A gene deletions and loss of p16 expression occur in osteosarcomas that lack RB alterations. *American Journal of Pathology*.

[37] Serena Benassi M, Molendini L, Gamberi G (1999). Alteration of prb/p16/cdk4 regulation in human osteosarcoma. *International Journal of Cancer*.

[38] Wei G, Lonardo F, Ueda T (1999). CDK4 gene amplification in osteosarcoma: reciprocal relationship with INK4A gene alterations and mapping of 12q13 amplicons. *International Journal of Cancer*.

[39] MacCarthy A, Bayne AM, Draper GJ (2009). Non-ocular tumours following retinoblastoma in Great Britain 1951 to 2004. *British Journal of Ophthalmology*.

[40] Wong FL, Boice JD, Abramson DH (1997). Cancer incidence after retinoblastoma: radiation dose and sarcoma risk. *JAMA*.

[41] Fletcher O, Easton D, Anderson K, Gilham C, Jay M, Peto J (2004). Lifetime risks of common cancers among retinoblastoma survivors. *Journal of the National Cancer Institute*.

[42] Fletcher O, Easton D, Anderson K, Gilham C, Jay M, Peto J (2004). Lifetime risks of common cancers among retinoblastoma survivors. *Journal of the National Cancer Institute*.

[43] MacCarthy A, Bayne AM, Draper GJ (2009). Non-ocular tumours following retinoblastoma in Great Britain 1951 to 2004. *British Journal of Ophthalmology*.

[44] Shields CL, Shields JA (2010). Retinoblastoma management: advances in enucleation, intravenous chemoreduction, and intra-arterial chemotherapy. *Current Opinion in Ophthalmology*.

[45] Patiño-García A, Sotillo Piñeiro E, Zalacaín Díez M, Gárate Iturriagagoitia L, Antillón Klüssmann F, Sierrasesúmaga Ariznabarreta L (2003). Genetic and epigenetic alterations of the cell cycle regulators and tumor suppressor genes in pediatric osteosarcomas. *Journal of Pediatric Hematology/Oncology*.

[46] Parulekar MV (2010). Retinoblastoma—current treatment and future direction. *Early Human Development*.

[47] Sheen V, Tucker MA, Abramson DH, Seddon JM, Kleinerman RA (2008). Cancer screening practices of adult survivors of retinoblastoma at risk of second cancers. *Cancer*.

[48] Serrano C, Alonso J, Gómez-Mariano G (2011). Low penetrance hereditary retinoblastoma in a family: what should we consider in the genetic counselling process and follow up?. *Familial Cancer*.

[49] Bruinooge SS (2003). American Society of Clinical Oncology policy statement update: genetic testing for cancer susceptibility. *Journal of Clinical Oncology*.

[50] Abramson DH, Beaverson K, Sangani P (2003). Screening for retinoblastoma: presenting signs as prognosticators of patient and ocular survival. *Pediatrics*.

[51] Sippel KC, Fraioli RE, Smith GD (1998). Frequency of somatic and germ-line mosaicism in retinoblastoma: implications for genetic counseling. *American Journal of Human Genetics*.

[52] Bohr VA (2008). Rising from the RecQ-age: the role of human RecQ helicases in genome maintenance. *Trends in Biochemical Sciences*.

[53] Monnat RJ (2010). Human RECQ helicases: roles in DNA metabolism, mutagenesis and cancer biology. *Seminars in Cancer Biology*.

[54] Wang LL, Levy ML, Lewis RA (2001). Clinical manifestations in a cohort of 41 Rothmund-Thomson syndrome patients. *American Journal of Medical Genetics*.

[55] Kitao S, Shimamoto A, Goto M (1999). Mutations in RECQL4 cause a subset of cases of Rothmund-Thomson syndrome. *Nature Genetics*.

[56] Larizza L, Roversi G, Volpi L (2010). Rothmund-thomson syndrome. *Orphanet Journal of Rare Diseases*.

[57] Macris MA, Krejci L, Bussen W, Shimamoto A, Sung P (2006). Biochemical characterization of the RECQ4 protein, mutated in Rothmund-Thomson syndrome. *DNA Repair*.

[58] Stinco G, Governatori G, Mattighello P, Patrone P (2008). Multiple cutaneous neoplasms in a patient with Rothmund-Thomson syndrome: case report and published work review. *The Journal of Dermatology*.

[59] Wang LL, Gannavarapu A, Kozinetz CA (2003). Association between osteosarcoma and deleterious mutations in the RECQL4 gene Rothmund-Thomson syndrome. *Journal of the National Cancer Institute*.

[60] Kääriäinen H, Ryoppy S, Norio R (1989). RAPADILIMO syndrome with radial and patellar aplasia/hypoplasia as main manifestations. *American Journal of Medical Genetics*.

[61] Siitonen HA, Kopra O, Kääriäinen H (2003). Molecular defect of RAPADILINO syndrome expands the phenotype spectrum of RECQL diseases. *Human Molecular Genetics*.

[62] Siitonen AH, Sotkasiira J, Biervliet M (2009). The mutation spectrum in RECQL4 diseases. *European Journal of Human Genetics*.

[63] Nakura J, Wijsman EM, Miki T (1994). Homozygosity mapping of the Werner syndrome locus (WRN). *Genomics*.

[64] Goto M (1997). Hierarchical deterioration of body systems in Werner’s syndrome: implications for normal ageing. *Mechanisms of Ageing and Development*.

[65] Yu CE, Oshima J, Fu YH (1996). Positional cloning of the Werner’s syndrome gene. *Science*.

[66] Goto M, Miller RW, Ishikawa Y, Sugano H (1996). Excess of rare cancers in Werner syndrome (adult progeria). *Cancer Epidemiology, Biomarkers & Prevention*.

[67] Ishikawa Y, Miller RW, Machinami R, Sugano H, Goto M (2000). Atypical osteosarcomas in Werner Syndrome (Adult Progeria). *Japanese Journal of Cancer Research*.

[68] Ellis NA, Groden J, Ye TZ (1995). The Bloom’s syndrome gene product is homologous to RecQ helicases. *Cell*.

[69] Ellis NA, Ciocci S, Proytcheva M, Lennon D, Groden J, German J (1998). The Ashkenazic Jewish bloom syndrome mutation blm(Ash) is present in non-Jewish Americans of Spanish ancestry. *American Journal of Human Genetics*.

[70] German J (1997). Bloom’s syndrome. XX. The first 100 cancers. *Cancer Genetics and Cytogenetics*.

[71] Li L, Eng C, Desnick RJ, German J, Ellis NA (1998). Carrier frequency of the Bloom syndrome blm(Ash) mutation in the Ashkenazi Jewish population. *Molecular Genetics and Metabolism*.

[72] Lipton JM, Atsidaftos E, Zyskind I, Vlachos A (2006). Improving clinical care and elucidating the pathophysiology of Diamond Blackfan anemia: an update from the Diamond Blackfan Anemia Registry. *Pediatric Blood and Cancer*.

[73] Vlachos A, Ball S, Dahl N (2008). Diagnosing and treating Diamond Blackfan anaemia: results of an international clinical consensus conference. *British Journal of Haematology*.

[74] Doherty L, Sheen MR, Vlachos A (2010). Ribosomal protein genes RPS10 and RPS26 are commonly mutated in Diamond-Blackfan anemia. *American Journal of Human Genetics*.

[75] Draptchinskaia N, Gustavsson P, Andersson B (1999). The gene encoding ribosomal protein S19 is mutated in Diamond-Blackfan anaemia. *Nature Genetics*.

[76] Lee RS, Higgs D, Haddo O, Pringle J, Briggs TWR (2004). Osteosarcoma associated with Diamond-Blackfan anaemia: a case of a child receiving growth hormone therapy. *Sarcoma*.

[77] Merks JHM, Caron HN, Hennekam RCM (2005). High incidence of malformation syndromes in a series of 1,073 children with cancer. *American Journal of Medical Genetics*.

[78] Villani A, Tabori U, Schiffman J (2011). Biochemical and imaging surveillance in germline TP53 mutation carriers with Li-Fraumeni syndrome: a prospective observational study. *The Lancet Oncology*.

